# Low-dose radiation decreases tumor progression via the inhibition of the JAK1/STAT3 signaling axis in breast cancer cell lines

**DOI:** 10.1038/srep43361

**Published:** 2017-02-27

**Authors:** Neha Kaushik, Min-Jung Kim, Rae-Kwon Kim, Nagendra Kumar Kaushik, Ki Moon Seong, Seon-Young Nam, Su-Jae Lee

**Affiliations:** 1 Department of Life Science, Research Institute for Natural Sciences, Hanyang University, Seoul 04763, Republic of Korea; 2 Laboratory of Radiation Exposure and Therapeutics, National Radiation Emergency Medical Center, Korea Institute of Radiological and Medical Sciences, Seoul, Korea; 3 The University of Texas MD Anderson Cancer Center, So Campus Research Bldg 1 (SCR2.2208), 1515 Holcombe Blvd. Unit 0903, Houston, TX 77030, USA; 4 Plasma Bioscience Research Center, Department of Electrical and Biological Physics, Kwangwoon University, Seoul, 139-701, Republic of Korea; 5 Radiation Health Institute, Korea Hydro and Nuclear Power Co. Ltd, Seoul, Korea

## Abstract

Breast cancer is a widely distributed type of cancer in women worldwide, and tumor relapse is the major cause of breast cancer death. In breast cancers, the acquisition of metastatic ability, which is responsible for tumor relapse and poor clinical outcomes, has been linked to the acquisition of the epithelial-mesenchymal transition (EMT) program and self-renewal traits (CSCs) via various signaling pathways. These phenomena confer resistance during current therapies, thus creating a major hurdle in radiotherapy/chemotherapy. The role of very low doses of radiation (LDR) in the context of EMT has not yet to be thoroughly explored. Here, we report that a 0.1 Gy radiation dose reduces cancer progression by deactivating the JAK1/STAT3 pathway. Furthermore, LDR exposure also reduces sphere formation and inhibits the self-renewal ability of breast cancer cells, resulting in an attenuated CD44^+^/CD24^−^ population. Additionally, *in vivo* findings support our data, providing evidence that LDR is a promising option for future treatment strategies to prevent cancer metastasis in breast cancer cases.

In nature, humans are regularly exposed to certain low doses of ionizing radiation, including medical radiotherapy, natural environmental background radiation and exposure to radioactive materials widely used in industrial applications. Therefore, examining the effects of low-dose radiation (LDR) has received a great deal of attention from those who study radiation biology. Nevertheless, there is also cumulative evidence indicating that radiation under certain doses could stimulate various repair mechanisms to reverse the initial damage and protect the organism from subsequent radiation or other exposures that may cause cancer^1^,^2^,^3^. The biological effects of LDR (<0.2 Gy) at certain levels differ from those of high-dose radiation (HDR; >2 Gy) in a manner which cannot be explained by the linear no-threshold (LNT) hypothesis. The LNT model means that the increased health risk is proportional to the received radiation dose at very low levels. This model implies that there is no threshold dose below which no increase in health risk is shown. For example, the chances of radiation-induced cancer in human survivors are greater for those exposed to higher doses (>2 Gy) relative to those exposed to low doses^4^. LDR exhibits various effects on organisms depending on the given dose rate and radiation rays used. While radiosensitivity levels differ considerably among individuals, the irradiation dose or frequency required to induce anticancer effects is also distinctive. Therefore, many issues must be clarified, such as the irradiation dose and the frequency to be used in clinical practice. One report suggests that a single dose of X-ray radiation represses tumor metastases in mice if given at low doses (0.1 or 0.2 Gy)^5^. It has also been shown that in mice exposed to single or fractionated low doses of X- or γ-rays, the growth of primary and/or metastatic tumors is delayed^6^,^7^. A total dose of 0.1 or 0.2 Gy applied fractionally was associated with less tumor formation in exposed mice models^8^. Moreover, LDR has been reported to improve the effectiveness of chemotherapeutic drugs^9^. Given that frequent tumor relapse after high-dose radiotherapy is the major cause of the failure of current treatment approaches and follow-up with the above mentioned beneficial effect of LDR, recently researchers are becoming more interested in checking epithelial-mesenchymal transition (EMT) processes with the use of LDR.

EMT is a well-recognized program is closely related to embryonic development and induced tumor progression^10^. The features of EMT involve losses of cell-cell contact and adhesion molecules, decreased expression of epithelial markers, and increased expression of mesenchymal markers^11^. Earlier data showed that breast tumor relapse after therapy is a hallmark of EMT^12^. Moreover, in breast cancer cases, the EMT state is linked to a cancer stem cell (CSC)-like population harboring the CD44+/CD24− profile, which shows resistance to therapies. The CSC-like population within cancer cells in particular has been proposed to play a critical role in metastatic progression and therapy resistance^13^.

In our previous work, we showed that LDR reduces the malignant phenotype in oncogene KRAS (Kirsten rat sarcoma viral oncogene homolog) transformed breast epithelial cells. This phenomenon is due to the LDR-induction of antioxidants counteracting KRAS-induced ROS levels in LDR-exposed cells^14^. As metastasis is associated with increased malignant tumor progression, we sought to examine the effects of LDR with regard to EMT in breast cancer cells. The data presented in this report indicate that LDR stimulates a decrease in cancer progression as induced by triple negative MDA-MB231 basal breast cancer cells. Moreover, down-regulation of JAK1/STAT3 phosphorylation confirms that these factors are critically involved in LDR-attenuated EMT and stemness in breast cancer cells. Using LDR along with an anticancer drug may be a more effective as well as the safest cancer treatment in future time.

## Results

### Diminution of CSC-like maintenance in breast cancer cells after LDR

**Figure 1:**
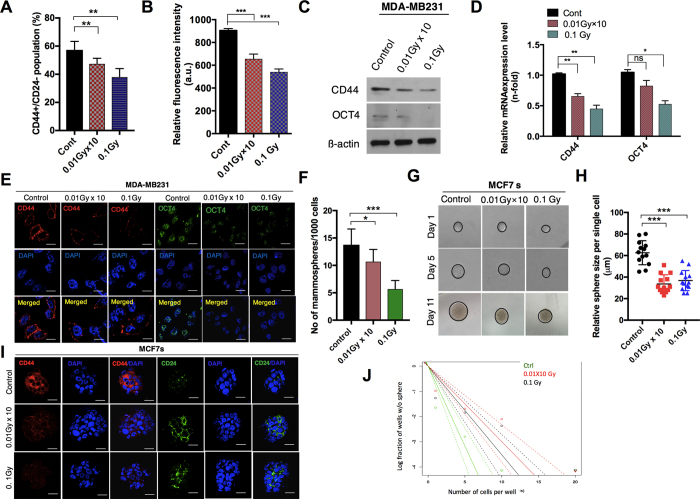
Low-dose radiation decreases the cancer stem-cell maintenance in breast cancer cell lines. (**A**) Flow cytometer analysis of CD44+/CD24− cells in control and LDR-exposed MDA-MB231 cells at 0.01 Gy × 10 (fractionated) and 0.1 Gy (single dose). (**B**) Determination of the CD44 fluorescence intensity in LDR treated and untreated control cells using an Elisa reader. (**C**) Western blot of the CD44 and OCT4 protein levels LDR treated and untreated control MDA-MB231 cells. (**D**) qRT-PCR analyses results of CD44 and OCT4 gene expression levels in LDR treated and untreated control MDA-MB231 cells. (**E**) Immunocytochemistry of CD24 and OCT4 expression levels in LDR treated and untreated control cells. (**F**) Determination of the sphere-forming ability of LDR-exposed and control MCF7 breast cancer cells cultured in a sphere-conditioned medium. (**G**,**H**) Single-cell assay of LDR-exposed and control MCF7 sphere cells as observed from days 1 to 11 days after treatment. Quantification of the average size of each single cell is shown in the representative graph. (**I**) Immunocytochemistry of CD24 and CD24 in LDR treated and untreated control MCF7 sphere-cultured cells. (**J**) Limiting dilution assay performed on MCF7 cells after LDR exposure and compared with LDR unexposed control cells. Solid lines represents the average value of samples. β-actin was used as a loading control. Error bars denote the mean ± S.D. of triplicate samples. *p < 0.05, **p < 0.01, and ***p < 0.001.

As cancer stem cells are highly responsible for tumor relapse after radiotherapy^15^, we initially focused on investigating the response of LDR in breast cancer cells with regard to CSC maintenance. To check the response of LDR in breast cancer, we initially examined a CD44+/CD24− population, i.e., those survived within breast cancer and maintained stemness, in LDR-exposed MDA-MB231 breast cancer cell lines. To this end, we used a dose of fractionated radiation (0.01 Gy ten times; 0.01 Gy × 10) and a single dose (0.1 Gy) to irradiate MDA-MB231 breast cancer cells. Flow cytometric and ELISA analyses revealed that LDR exposure attenuated the CD44+/24− cell population in MDA-MB231 cells (Fig. 1A,B). Consistent with this data, CD44 protein levels were also found to be reduced after LDR exposure in these cell lines (Fig. 1C). Apart from CD44, cancer stem cells displayed high levels of the OCT4 levels^16^. When the levels of these proteins were analyzed in LDR-exposed MDA-MB231 cells, it was found that LDR reduced the protein levels of OCT4 remarkably to a greater extent (Fig. 1C). The reduced OCT4 and CD44 expression confirmed our findings that LDR is capable of diminishing CSC-like population in MDA-MB231 triple-negative breast cancer cells (Fig. 1D). In agreement with these results, immunofluorescence staining confirmed that CD44 and OCT4 expression levels were noticeably decreased after LDR exposure in MDA-MB231 cells (Fig. 1E). To mimic the basal phenotype, we further examined LDR-exposed MCF7 breast cancer cells in a sphere-condition medium. A subpopulation of breast cancer cells has been demonstrated to have stem-like cell properties, such as self-renewal or sphere-forming capabilities^17^,^18^. To check whether LDR can have an effect on stem-cell-like characteristics in breast cancer, we utilized sphere clonal assays. Notably, we observed a significant reduction in sphere formation in MCF7 cells after LDR exposure (Fig. 1F). Moreover, a single cell assay showed that the sphere size was also smaller after LDR exposure in MCF7 cells when compared to a control (Fig. 1G,H). Broadly, CD44+/CD24− population profile is recognized in breast cancer for the presence of tumor initiating cells. To this end, CD44+CD24− staining further supported our observations that LDR can modulate these populations in breast cancer (Fig. 1I). We also demonstrated that the degree of sphere formation was reduced after LDR treatment in sphere-cultured MCF7 breast cancer cells, as confirmed by a limiting dilution assay (Fig. 1J). Taken together, these results indicate that LDR has the potential to reduce the maintenance of CSC-like populations in breast cancer cells.

### LDR suppresses the invasiveness of breast cancer cells

**Figure 2:**
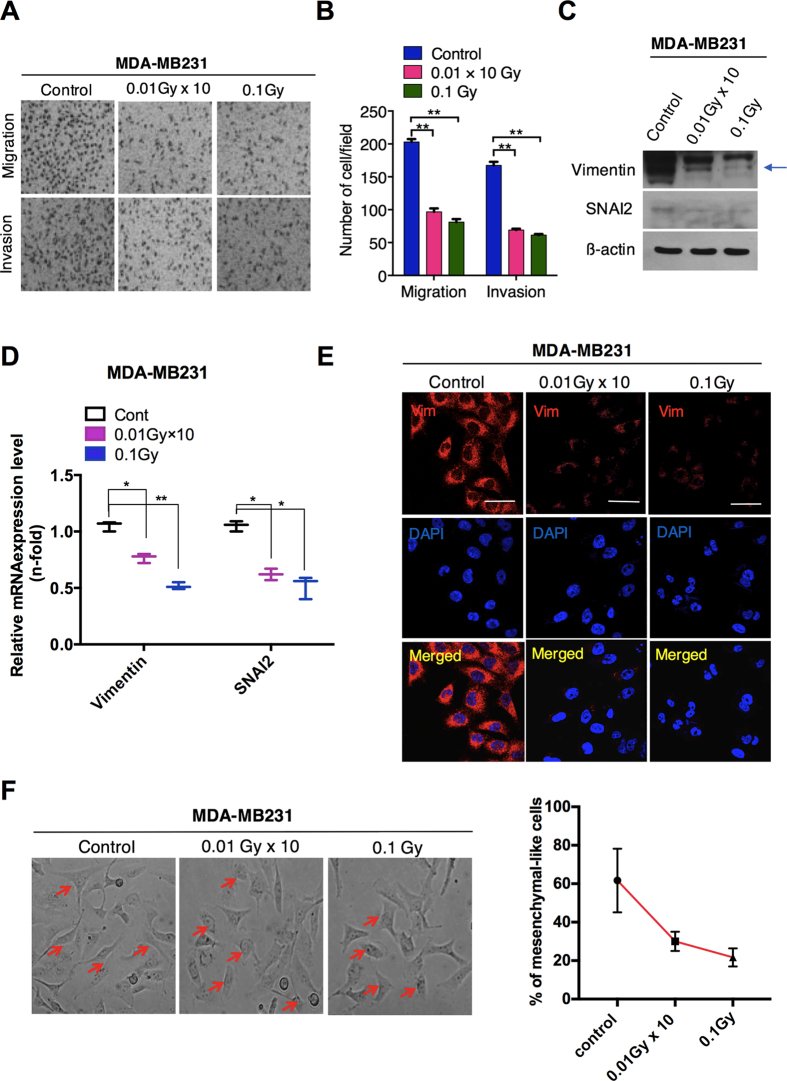
Low-dose radiation reduces the migration and invasion in MDA-MB231 cells via EMT. (**A**) Migration and invasion Transwell assays of MDA-MB231 breast cancer cells after LDR at doses of 0.01 Gy × 10 (fractionated) and 0.1 Gy (single dose). (**B**) Representative graphs of the migration and invasion of LDR-exposed MDA-MB231 cells. (**C**) Western blot for vimentin and SNAI2 (SLUG) in MDA-MB231 breast cancer cells after irradiation at 0.01Gy × 10 and 0.1Gy. (**D**) qRT-PCR analysis of vimentin and SNAI2 gene expression after irradiation in MDA-MB231 cells. (**E**) Immunocytochemistry for a vimentin mesenchymal marker in MDA-MB231 breast cancer cells after irradiation. (**F**) Phase-contrast images of control and LDR-treated MDA-MB231 cells. Representative graph shows the percentage of mesenchymal-like cells in respective each group. β-actin was used as a loading control. Error bars denote the mean ± S.D. of triplicate samples. *p < 0.05, and **p < 0.01.

We subsequently assessed whether LDR can inhibit tumor progression via the EMT process, which is characterized by increased cellular motility and invasiveness along with a loss of epithelial markers. Our data showed that a single or fractionated dose (0.1 Gy) of LDR decreased migration and invasion in MDA-MB231 cells (Fig. 2A,B). Notably, EMT marker and transcription factor changes, including the reduced vimentin and SNAI2 levels, were observed after the cells were treated with LDR (Fig. 2C), indicating decreased EMT process in MDA-MB231 cells. Moreover, the expressions of vimentin and SNAI2 were significantly downregulated after LDR exposure in these breast cancer cells (Fig. 2D). In addition, less accumulation of vimentin on cell membranes was noted in LDR-exposed cells as compared to control cells (Fig. 2E). A morphological analysis revealed that cells undergoing a LDR treatment became shorter and rounder, as shown in Fig. 2F. Collectively, we provide strong evidence that LDR is capable of suppressing the EMT-like phenomenon in MDA-MB231 breast cancer cells.

### LDR blocks CSCs and invasiveness by inhibiting JAK1/STAT3 signaling in breast cancer cells

**Figure 3:**
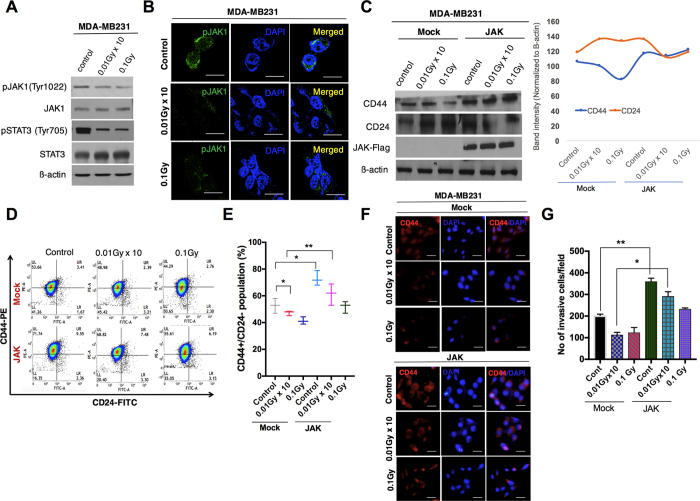
Low-dose radiation inhibits JAK1/STAT3 signaling in breast cancer cells. (**A**) Western blot analysis for the phosphorylation status of the JAK1/STAT3 pathway in LDR-exposed MDA-MB231 cells. (**B**) Immunofluorescence of the pJAK1 expression status in LDR-treated MDA-MB231 cells at similar doses. (**C**) Western blot analyses of the protein levels of CD24 and CD44 in LDR-irradiated MDA-MB231 cells after JAK1 overexpression. Representative graphs show the intensity of the CD44/CD24 levels in the LDR-exposed MDA-MB231 cells. (**D**) Flow cytometer analysis of CD44+CD24− cells in control and JAK1-overexpressing LDR-exposed MDA-MB231 cells at 0.01Gy × 10 (fractionated) and 0.1Gy (single dose). (**E**) Representative graph of the CD44+CD24− population in Mock and JAK1-overexpressing LDR-irradiated cells at similar doses. (**F**) Immunofluorescence staining of the CD44 expression outcomes in LDR-exposed MDA-MB231 cells after JAK1 overexpression. (**G**) Analysis of invasive cells in LDR-irradiated JAK1-overexpressing MDA-MB231 cells. β-actin was used as a loading control. Error bars denote the mean ± S.D. of triplicate samples. *p < 0.05, and **p < 0.01.

We then sought to determine the signaling mechanism downregulating CSC and EMT in response to radiation. It has been reported that the JAK/STAT signaling pathway is required for the maintenance of CD44^+^ CD24^−^ stem-cell-like breast cancer cells in cancer cells^19^,^20^. In line with these studies, we checked the phosphorylation status of the JAK/STAT pathway in LDR-exposed MDA-MB231 cells. Our data demonstrated that the phosphorylation of JAK1 and STAT3 was decreased more pronouncedly in LDR-exposed cells as compared to a control (Fig. 3A). Additionally, immunofluorescence staining data confirmed that pJAK1 was noticeably decreased in MDA-MB231 cells after LDR exposure (Fig. 3B). As noted above, as the JAK/STAT pathway is crucial for CSC maintenance, we checked the protein levels of CD44 and CD24 in LDR-exposed MDA-MB231 cells after JAK1 overexpression. We observed that JAK overexpression increased the CD44 and CD24 levels in those LDR-treated cells compared to a control (Fig. 3C). We also checked for CD44 positive cells in JAK-overexpressing MDA-MB231 LDR cells. Interestingly, we found a significant increase in the CD44 population in LDR-exposed JAK-overexpressing MDA-MB231 cells when compared to a control (Fig. 3D,E). Moreover, immunofluorescence data provide support for our observations that JAK overexpression enhances the expression of CD44 in LDR-exposed breast cancer cells as compared to a control (Fig. 3F). A considerable amount of evidence suggests that the JAK/STAT pathway regulates the EMT process in various cancer cells^21^. To this end, we subsequently measured the involvement of this pathway with regard to EMT in the response to radiation. As expected, LDR significantly attenuated migration and invasion in JAK-overexpressing basal-type MDA-MB231 cells (Fig. 3G). Taken together, we conclude here that LDR halts the invasiveness in breast cancer cells via the attenuation of CSC maintenance and EMT.

### LDR suppresses lung metastasis by breast cancer cells

**Figure 4:**
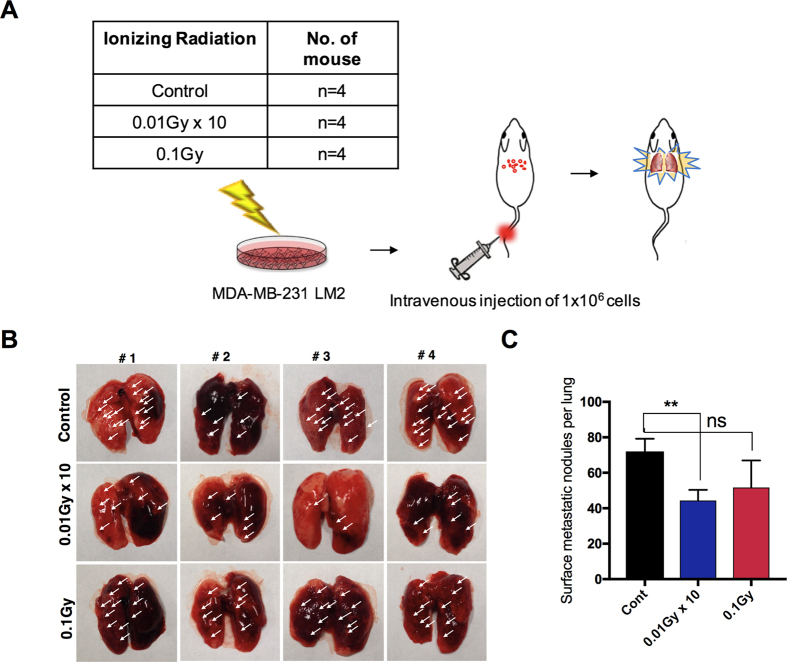
Low-dose radiation reduces breast metastasis to the lungs. (**A**) Schematic plan of LDR irradiation in mice models (n = 5 each group). GFP-tagged metastatic MDA-MB231 LM2 cells were injected through the tail vein. (**B**) Representative images and (**C**) quantification of the surface metastatic foci in the lungs of LDR-exposed mice. Error bars represent the mean ± S.D. of triplicate samples. *p < 0.05, and **p < 0.01.

Given that our data as described above revealed that LDR reduces invasiveness through EMT, we decided to determine whether our findings are also valid *in vivo*. To test whether LDR can decrease breast metastasis *in vivo*, metastatic MDA-MB231 LM2 breast cancer cells were transplanted into the tail vein of athymic balb/c nude mice (n = 4) (Fig. 4A). Compared to a control, lung metastasis was markedly decreased in LDR-treated tumors than in the control cases. Of note, the number of metastatic foci was effectively decreased in the LDR-treated mice compared to their control counterparts (Fig. 4B,C). Collectively, these results suggest that LDR reduces the metastatic ability of breast cancer cells *in vivo* to the lungs.

## Materials & Methods

### Cell culture and antibodies

Breast cancer cell lines MCF-7 and MDA-MB231 were established from the American Type Culture Collection (Manassas, VA). MCF7 cells were grown in MEM and MDA-MB231 cells were grown in DMEM. All media were supplemented with 10% fetal bovine serum, penicillin (100 units/ml), and streptomycin (100 g/ml). These cells were cultured in a humidified 5% CO_2_ atmosphere at 37 °C. To grow breast cancer cells under a sphere culture condition, cells were cultured in serum-free DMEM-F12 media (Invitrogen, Korea) as described previously^22^. Antibodies specific to OCT4, SLUG (SNAI2), pJAK1(Tyr1022), JAK1, and STAT3 were obtained from Santa Cruz Biotechnology, Inc. CD44-PE and CD24-FITC antibodies were purchased from BD Transduction Laboratory (Seoul, Korea). Antibodies to pSTAT3 (Tyr705) were purchased from Cell Signaling Technology. Vimentin and CD44 were purchased from Abcam (Cambridge, UK), whereas 4,6-Diamidino-2-phenylindole (DAPI), an antibody specific to Flag and β-actin were obtained from Sigma, Korea. Anti-mouse or rabbit Alexa Fluor 488 and anti-rabbit or mouse Alexa Fluor 546 were purchased from Invitrogen.

### Irradiation

Breast cancer cells were plated in 60-mm dishes and irradiated with a 137Cs laboratory γ-irradiator (LDI-KCCH 137, Seoul, Korea) at a dose rate of 0.1 Gy/min for the time required to apply a prescribed dose at room temperature. For fractionated radiation of 0.1 Gy, cells were irradiated on five consecutive days per week for two weeks to establish 0.01 Gy × 10 fractionated radiation cells. The single dose of radiation (0.1 Gy) was applied at the same time as the last dose of the fractionated radiation.

### Transfection

JAK1 overexpression was performed by transfecting the expression plasmid (Myc-DDK-tagged)-Human Janus kinase 1 (Origene Technologies, Rockville, MD, USA) using the transfection reagent Lipofectamine (Invitrogen Corp.). The empty vector was used as the vehicle control.

### Flow cytometry

To examine the breast cancer stem cell population with enriched CD44 expression, the LDR-exposed 5 × 10^5^ breast cancer cells were labeled with an anti-CD44/PE antibody (BD Biosciences, Korea). A respective control was also prepared and tested for each sample. Briefly, control and exposed cells were incubated for 20–25 min at 4 °C, washed twice with PBS, and immediately analyzed using a BD FACSVerse cytometer and the FACS suite software. The percentage of dead cells was determined using propidium iodide (PI, 50 ng/ml) (Sigma) according to the manufacturer’s instructions and was analyzed using a flow cytometer.

### Migration and invasion assays

To assess the degrees of the migration and invasion, 2 × 10^4^ cells were plated in Transwell plates. For the invasion experiment, we used chambers with inserts that were pre-coated with growth-factor-reduced Matrigel (BD Biosciences), however; the inserts left uncoated so as to undertake the migration assays as described previously^23^.

### Self-renewal assays

Briefly, for the sphere-forming assays, LDR exposed MCF7 sphere cells were collected and dissociated into single cells and further plated in an ultra-low-attachment 60mm dish at 1 × 10^3^ cells/ml in triplicate. The numbers of spheres which formed in the control and LDR-exposed breast cancer cells were monitored on days 1–4 using Motic Images Plus 2.0 software in three randomly chosen fields. For the self-renewal or clonal assays, sphere cells were dissociated using Accutase and individual breast cancer sphere cells were plated into 96-well plates. On the next day, each well was visually checked to verify the presence of a single cell. After the clones were grown, clone formation was monitored for several days. Clone sizes were measured using a phase-contrast microscope with the Motic Images Plus 2.0 software^24^.

### Limiting dilution assays

Briefly, cells were plated at different dilutions, in this case 1 × 10^3^ cells/ml, 4 × 10^2^ cells/ml, 2 × 10^2^ cells/ml, and 1 × 10^2^ cells/ml for 16, 32, 48, and 96 wells, respectively, in ultra-low-attachment 96-well cell-culture plates. Data were analyzed using the online tool available at http://bioinf.wehi.edu.au/software/elda/.

### Immunoblotting

Cell lysates were prepared by extracting proteins with lysis buffer [40 mM Tris-HCl (pH 8.0), 120 mM NaCl, 0.1% Nonidet-P40] supplemented with protease inhibitors. Proteins were separated by SDS-PAGE and then transferred to a nitrocellulose membrane (Amersham, IL). Membranes were blocked with 5% skim milk in Tris-buffered saline and incubated with primary antibodies overnight at 4 °C. The blots were then developed with a peroxidase-conjugated secondary antibody, and the proteins were visualized by enhanced chemiluminescence (ECL) procedures (Amersham, IL) using the manufacturer’s protocol.

### Immunofluorescence

Briefly, cells were fixed with 4% paraformaldehyde and permeabilized with 0.1% Triton X-100 in PBS. Following cell fixation, cells were incubated with the appropriate primary antibodies in a solution of PBS with 1% bovine serum albumin and 0.1% Triton X-100 at 4 °C overnight. Human anti-CD44 (anti-rabbit, 1:200), OCT4 (anti-rabbit, 1:200), and vimentin (anti-mouse, 1:200), pJAK1 (anti-rabbit, 1:200) and CD24-FITC (1:200) were used. Staining was visualized using anti-rabbit or anti-mouse Alexa Flour 488 and anti-rabbit or anti-mouse Alexa Flour 546 (molecular probe) antibodies, and nuclei were counterstained using 4,6-diamidino-2-phenylindole (DAPI; Sigma). These cells were imaged with a confocal fluorescence microscope (Nikon).

### Real time PCR

Total RNA was extracted using the TRIzol reagent (Ambion). All qRT-PCR processes were accomplished using the KAPA SYBR FAST qPCR kit from KAPA Biosystems (Wilmington, MA, USA) according to the manufacturer’s instructions. Amplification reactions were carried out in a Rotor Gene Q (Qiagen, Korea), and results were expressed as the fold change calculated by the ΔΔCt method relative to the control sample. β-actin was used for normalization as a control.

### Animal experiments

GFP-tagged metastatic MDA-MB231 LM2 breast cancer cells (1 × 10^6^ cells/200 μl PBS) were intravenously injected into the tail of athymic BALB/c female nude mice (7–8 weeks of age; Orient). Once tumors had reached to 100 mm^3^, the mice were randomly divided into LDR irradiation and control groups. Lung metastasis was analyzed by counting the number of surface metastatic foci in the lung in both groups. This study was reviewed and approved by the Institutional Animal Care and Use Committee (IACUC) of the Center for Laboratory Animal Sciences, the Medical Research Coordinating Center, and the HYU Industry-University Cooperation Foundation. All methods were performed in accordance with the relevant guidelines and regulations.

### Statistical analysis

All experimental data are represented as the mean ± standard deviation (S.D.) of at least three independent tests. The statistical analysis was performed using the parametric Student’s *t*-test to check the significance levels.

## Discussion

Breast cancer is a deadly form of cancer among women worldwide^25^. Consequently, advances in the treatment of this cancer have high priority among researchers. In recent years, radiotherapy has been widely used to treat various cancers effectively at advanced stages. However, the acquisition of radioresistance during radiotherapy in patients causes therapy to become ineffective^26^. Apart from this, damage to normal tissues is unavoidable during high-dose radiation treatments. A successful radiation therapy is considered as an enhancement of tumor cell death through the activation of tumor immunogenicity while simultaneously minimizing the adverse effects on surrounding healthy tissues. Georgakilas *et al*.^27^ recently suggested that with low and high doses of radiation, it is very difficult to predict the responses of all signaling pathways and inflammatory factors uniformly for all types of radiation doses given. On the other hand, it is also well established that radiation exposure induces changes in the immune system through the induction of an inflammatory environment and radiation-induced cell death^28^. Reasonable X-rays below 1 Gy, i.e., at very low doses of ionizing radiation, can have certain anti-inflammatory events while high doses mainly cause cellular DNA and protein damage, eventually resulting in radiation-induced immune modulation^29^. Hence, it is challenging to distinguish between the different means of radiation at effective doses without, or with less harm to, the surrounding tissues for those in the radio therapeutic field. Here, our results showed that LDR decreased malignancy by suppressing EMT and cancer stem-cell maintenance in breast cancer cells.

Many reports have provided evidence that EMT is responsible for boosting cell motility and invasiveness in multicellular organisms^30^, promoting cancer development^31^. Here, we report that LDR decreases the migration and invasion in triple-negative MDA-MB231 breast cancer cells. Moreover, LDR downregulated the expression levels of vimentin and SNAI2 (SLUG) (Fig. 2), critical markers of EMT regulation, more prominently. All of these markers of tumor progression have been held to be associated with higher CSC maintenance. This CSC population can be prolonged by the EMT process^32^. It has been reported that breast cancer cells with the CD44+CD24– phenotype and stem-cell–like features are highly resistant to cancer therapies^33^. Here, our data demonstrate that LDR exposure leads to a reduction of the CD44 population with the subsequent ablation of CSC-like features, such as self-renewal. Our findings also suggest that LDR reduces the clonal frequency in breast cancer cells, as confirmed with limiting dilution assays (Fig. 1).

The JAK/STAT pathway has been thoroughly explored in relation to various cancer types^34^. The constitutive activation of STAT3 signaling due to aberrations in JAK has been confirmed in various hematopoietic malignancies^35^. Following up on these studies, recently Marotta *et al*. claimed that the JAK2/STAT3 signaling pathway is required for the growth of CD44+CD24– stem-cell–like breast cancer cells in human tumors^19^. Generally, CD44+CD24– cells are relatively high in incidences of basal-like breast cancer as compared to luminal tumor cells^36^,^37^. In agreement with these studies, we propose here that LDR downregulates the JAK1/STAT3 pathway more noticeably in MDA-MB231 cells. Moreover, JAK overexpression reverses the effect of LDR on migration and invasiveness along with CSC maintenance.

In summary, we demonstrated that LDR effectively suppresses the markers and regulator levels that are specifically required for the EMT program. A similar effect was observed in the case of the maintenance of CD44+CD24− breast cancer cells by LDR. Inhibition of the JAK1/STAT3 pathway is crucial for targeting aggressive characteristics in these basal like cells. Furthermore, *in vivo* findings suggested that LDR has the potential to suppress lung metastases of breast cancer cells. Fractionated as well as single doses of LDR effectively decrease the lung metastatic nodules; however, fractionated radiation more significantly suppresses lung metastasis. This may arise because a single dose imparts more stress on an animal at one time, whereas fractionated doses do not present strong stress with each given dose as compared to a single-dose treatment. It may be that a single dose of radiation stimulates cross-priming, resulting in a lack of control of “bulky” palpable tumors. Collectively, our data suggested that the use of LDR is a promising approach for the safe treatment of breast cancer at low doses.

## Additional Information

**How to cite this article**: Kaushik, N. *et al*. Low-dose radiation decreases tumor progression via the inhibition of the JAK1/STAT3 signaling axis in breast cancer cell lines. *Sci. Rep.* **7**, 43361; doi: 10.1038/srep43361 (2017).

**Publisher's note:** Springer Nature remains neutral with regard to jurisdictional claims in published maps and institutional affiliations.

## References

[b1] Wei L. X., Zha Y. R. & Tao Z. F. Epidemiological investigation of radiological effects in high background radiation areas of Yangjiang, China. J Radiat Res, J Radiat Res. 31, 119–36 (1990). 2141080

[b2] Lehrer S. & Rosenzweig K. E. Lung cancer hormesis in high impact states where nuclear testing occurred. Clin Lung Cancer. 16, 152–5 (2015). 25458560 10.1016/j.cllc.2014.09.010 PMC6587186

[b3] Nambi K. S. & Soman S. D. Environmental radiation and cancer in India. Health Phys. 52, 653–7 (1987). 3570803 10.1097/00004032-198705000-00018

[b4] Kamiya K. . Long-term effects of radiation exposure on health. Lancet. 386, 469–78 (2015) 26251392 10.1016/S0140-6736(15)61167-9

[b5] Cheda A. . Single low doses of X rays inhibit the development of experimental tumor metastases and trigger the activities of NK cells in mice. Radiat Res. 161, 335–40 (2004). 14982480 10.1667/rr3123

[b6] Hosoi Y. & Sakamoto K. Suppressive effect of low dose total body irradiation on lung metastasis: dose dependency and effective period. Radiother Oncol. 26, 177–9 (1993). 8465019 10.1016/0167-8140(93)90101-d

[b7] Hashimoto S. . The suppression of metastases and the change in host immune response after low-dose total-body irradiation in tumor-bearing rats. Radiat Res. 151, 717–24 (1999). 10360792

[b8] Nowosielska E. M., Cheda A., Wrembel-Wargocka J. & Janiak M. K. Modulation of the growth of pulmonary tumour colonies in mice after single or fractionated low-level irradiations with X-rays. Nukleonika. 53, S9–S15 (2008).

[b9] Zhang Y., Lu Z. & Li X. Y. Effect of combined whole-body low dose irradiation and chemotherapy on growth, metastasis and immune functions in tumor bearing mice. Radiate Prot. 19, 127–131 (1999)

[b10] Lee M. Y. & Shen M. R. Epithelial-mesenchymal transition in cervical carcinoma. Am J Transl Res. 4, 1–13 (2012). 22347518 PMC3276374

[b11] Zeisberg M. & Neilson E. G. Biomarkers for epithelial-mesenchymal transitions. J Clin Invest. 119, 1429–1437 (2009). 19487819 10.1172/JCI36183 PMC2689132

[b12] Moody S. E. . The transcriptional repressor Snail promotes mammary tumor recurrence. Cancer Cell. 8, 197–209 (2005). 16169465 10.1016/j.ccr.2005.07.009

[b13] Liu S. & Wicha M. S. Targeting breast cancer stem cells. J Clin Oncol. 28, 4006–12 (2010). 20498387 10.1200/JCO.2009.27.5388 PMC4872314

[b14] Kim R. K. . Beneficial effects of low dose radiation in response to the oncogenic KRAS induced cellular transformation. Sci. Rep. 5, 15809 (2015). 26515758 10.1038/srep15809 PMC4626770

[b15] Mitra A., Mishra L. & Li S. EMT, CTCs and CSCs in tumor relapse and drug-resistance. Oncotarget. 6, 10697–10711(2015). 25986923 10.18632/oncotarget.4037 PMC4484413

[b16] Koo B. S. . Oct4 is a critical regulator of stemness in head and neck squamous carcinoma cells. Oncogene. 34, 2317–2 (2014). 24954502 10.1038/onc.2014.174

[b17] Al-Hajj M., Wicha M. S., Benito-Hernandez A., Morrison S. J. & Clarke M. F. Prospective identification of tumorigenic breast cancer cells. Proc Natl Acad Sci USA 100, 3983–3988 (2003). 12629218 10.1073/pnas.0530291100 PMC153034

[b18] Sheridan C. . CD44+/CD24− breast cancer cells exhibit enhanced invasive properties: an early step necessary for metastasis. Breast Cancer Res. 8, R59 (2006). 17062128 10.1186/bcr1610 PMC1779499

[b19] Marotta L. L. . The JAK2/STAT3 signaling pathway is required for growth of CD44^+^ CD24^−^ stem cell-like breast cancer cells in human tumors. J Clin Invest. 121, 2723–35 (2011) 21633165 10.1172/JCI44745 PMC3223826

[b20] So J. Y. . Targeting CD44-STAT3 signaling by Gemini vitamin D analog leads to inhibition of invasion in basal-like breast cancer. PLoS One. 8, e54020 (2013). 23326564 10.1371/journal.pone.0054020 PMC3543376

[b21] Kim M. S., Lee W. S., Jeong J., Kim S. J. & Jin W. Induction of metastatic potential by TrkB via activation of IL6/JAK2/STAT3 and PI3K/AKT signaling in breast cancer. Oncotarget. 6, 40158–71 (2015). 26515594 10.18632/oncotarget.5522 PMC4741886

[b22] Kim R. K., Uddin N., Hyun J. W., Kim C., Suh Y. & Lee S. J. Novel anticancer activity of phloroglucinol against breast cancer stem-like cells. Toxicol Appl Pharmacol. 286, 143–50 (2015). 25843036 10.1016/j.taap.2015.03.026

[b23] Kaushik N. K. . Low doses of PEG-coated gold nanoparticles sensitize solid tumors to cold plasma by blocking the PI3K/AKT-driven signaling axis to suppress cellular transformation by inhibiting growth and EMT. Biomaterials. 87, 118–30 (2016). 26921841 10.1016/j.biomaterials.2016.02.014

[b24] Lu F. & Wong C. S. A clonogenic survival assay of neural stem cells in rat spinal cord after exposure to ionizing radiation. Radiat. Res. 163, 63–71 (2005). 15606308 10.1667/rr3285

[b25] Senkus E. . Primary breast cancer: ESMO Clinical Practice Guidelines for diagnosis, treatment and follow-up. Ann Oncol . 26 Suppl 5, v8–v30 (2015). 10.1093/annonc/mdv298 26314782

[b26] Kitahara O., Katagiri T., Tsunoda T., Harima Y. & Nakamura Y. Classification of sensitivity or resistance of cervical cancers to ionizing radiation according to expression profiles of 62 genes selected by cDNA microarray analysis. Neoplasia. 4, 295–303 (2002). 12082545 10.1038/sj.neo.7900251 PMC1531706

[b27] Georgakilas A. G. . Emerging molecular networks common in ionizing radiation, immune and inflammatory responses by employing bioinformatics approaches. Cancer Lett. 368, 164–72 (2015). 25841996 10.1016/j.canlet.2015.03.021

[b28] Candéias S. M., Mancini S. J., Touvrey C., Borel E., Jouvin-Marche E. & Marche P. N. p53-dependent and p53-independent pathways for radiation-induced immature thymocyte differentiation. Oncogene 23, 1922–1929 (2004). 14755249 10.1038/sj.onc.1207320

[b29] Rödel F., Frey B., Multhoff G. & Gaipl U. Contribution of the immune system to bystander and non-targeted effects of ionizing radiation. Cancer Lett 356, 105–113 (2015). 24139966 10.1016/j.canlet.2013.09.015

[b30] Thiery J. P. Epithelial-mesenchymal transitions in development and pathologies. Curr Opin Cell Biol. 15, 740–746 (2003). 14644200 10.1016/j.ceb.2003.10.006

[b31] Lee M. Y., Chou C. Y., Tang M. J. & Shen M. R. Epithelial mesenchymal transition in cervical cancer: correlation with tumor progression, epidermal growth factor receptor overexpression and snail up-regulation. Clin Cancer Res. 14, 4743–4750 (2008). 18676743 10.1158/1078-0432.CCR-08-0234

[b32] Scheel C. & Weinberg R. A. Cancer stem cells and epithelial-mesenchymal transition: concepts and molecular links. Semin Cancer Biol. 22, 396–403 (2012). 22554795 10.1016/j.semcancer.2012.04.001 PMC6220425

[b33] Frank N. Y., Schatton T. & Frank M. H. The therapeutic promise of the cancer stem cell concept. J Clin Invest. 120, 41–50 (2010). 20051635 10.1172/JCI41004 PMC2798700

[b34] Bollrath J. & Greten F. R. IKK/NF-kappaB and STAT3 pathways: central signalling hubs in inflammation-mediated tumour promotion and metastasis. EMBO Rep. 10, 1314–9 (2009). 19893576 10.1038/embor.2009.243 PMC2799209

[b35] Vainchenker W., Dusa A. & Constantinescu S. N. AKs in pathology: role of Janus kinases in hematopoietic malignancies and immunodeficiencies. Semin Cell Dev Biol. 19, 385–93 (2008). 18682296 10.1016/j.semcdb.2008.07.002

[b36] Honeth G. . The CD44+/CD24− phenotype is enriched in basal-like breast tumors. Breast Cancer Res. 10, R53, doi: 10.1186/bcr2108 (2008). 18559090 PMC2481503

[b37] Park S. Y. . Heterogeneity for stem cell-related markers according to tumor subtype and histologic stage in breast cancer. Clin Cancer Res. 16, 876–887 (2010). 20103682 10.1158/1078-0432.CCR-09-1532 PMC2818503

